# Evaluation of Excess Significance Bias in Animal Studies of Neurological Diseases

**DOI:** 10.1371/journal.pbio.1001609

**Published:** 2013-07-16

**Authors:** Konstantinos K. Tsilidis, Orestis A. Panagiotou, Emily S. Sena, Eleni Aretouli, Evangelos Evangelou, David W. Howells, Rustam Al-Shahi Salman, Malcolm R. Macleod, John P. A. Ioannidis

**Affiliations:** 1 Department of Hygiene and Epidemiology, University of Ioannina School of Medicine, Ioannina, Greece; 2 Department of Clinical Neurosciences, University of Edinburgh, Edinburgh, United Kingdom; 3 The Florey Institute of Neuroscience and Mental Health, University of Melbourne, Heidelberg, Victoria, Australia; 4 Department of Methods and Experimental Psychology, University of Deusto, Bilbao, Spain; 5 Laboratory of Cognitive Neuroscience, School of Psychology, Aristotle University of Thessaloniki, Thessaloniki, Greece; 6 Stanford Prevention Research Center, Department of Medicine, and Department of Health Research and Policy, Stanford University School of Medicine, and Department of Statistics, Stanford University School of Humanities and Sciences, Stanford, California, United States of America; University of California San Francisco, United States of America

## Abstract

Animal studies generate valuable hypotheses that lead to the conduct of preventive or therapeutic clinical trials. We assessed whether there is evidence for excess statistical significance in results of animal studies on neurological disorders, suggesting biases. We used data from meta-analyses of interventions deposited in Collaborative Approach to Meta-Analysis and Review of Animal Data in Experimental Studies (CAMARADES). The number of observed studies with statistically significant results (O) was compared with the expected number (E), based on the statistical power of each study under different assumptions for the plausible effect size. We assessed 4,445 datasets synthesized in 160 meta-analyses on Alzheimer disease (*n* = 2), experimental autoimmune encephalomyelitis (*n* = 34), focal ischemia (*n* = 16), intracerebral hemorrhage (*n* = 61), Parkinson disease (*n* = 45), and spinal cord injury (*n* = 2). 112 meta-analyses (70%) found nominally (*p*≤0.05) statistically significant summary fixed effects. Assuming the effect size in the most precise study to be a plausible effect, 919 out of 4,445 nominally significant results were expected versus 1,719 observed (*p*<10^−9^). Excess significance was present across all neurological disorders, in all subgroups defined by methodological characteristics, and also according to alternative plausible effects. Asymmetry tests also showed evidence of small-study effects in 74 (46%) meta-analyses. Significantly effective interventions with more than 500 animals, and no hints of bias were seen in eight (5%) meta-analyses. Overall, there are too many animal studies with statistically significant results in the literature of neurological disorders. This observation suggests strong biases, with selective analysis and outcome reporting biases being plausible explanations, and provides novel evidence on how these biases might influence the whole research domain of neurological animal literature.

## Abstract 2

The evaluation of 160 meta-analyses of animal studies on potential treatments for neurological disorders reveals that the number of statistically significant results was too large to be true, suggesting biases.

## Author Summary

Studies have shown that the results of animal biomedical experiments fail to translate into human clinical trials; this could be attributed either to real differences in the underlying biology between humans and animals, to shortcomings in the experimental design, or to bias in the reporting of results from the animal studies. We use a statistical technique to evaluate whether the number of published animal studies with “positive” (statistically significant) results is too large to be true. We assess 4,445 animal studies for 160 candidate treatments of neurological disorders, and observe that 1,719 of them have a “positive” result, whereas only 919 studies would a priori be expected to have such a result. According to our methodology, only eight of the 160 evaluated treatments should have been subsequently tested in humans. In summary, we judge that there are too many animal studies with “positive” results in the neurological disorder literature, and we discuss the reasons and potential remedies for this phenomenon.

## Introduction

Animal research studies make a valuable contribution in the generation of hypotheses that might be tested in preventative or therapeutic clinical trials of new interventions. These data may establish that there is a reasonable prospect of efficacy in human disease, which justifies the risk to trial participants.

Several empirical evaluations of the preclinical animal literature have shown limited concordance between treatment effects in animal experiments and subsequent clinical trials in humans [1]–[4]. Systematic assessments of the quality of animal studies have attributed this translational failure, at least in part, to shortcomings in experimental design and in the reporting of results [5]. Lack of randomization, blinding, inadequate application of inclusion and exclusion criteria, inadequate statistical power, and inappropriate statistical analysis may compromise internal validity [6],[7].

These problems are compounded by different types of reporting biases [8]. First, bias against publication of “negative” results (publication bias) or publication after considerable delay (time lag bias) may exist [9]. Such findings may not be published at all, published with considerable delay, or published in low impact or low visibility national journals in comparison to studies with “positive” findings. Second, selective analysis and outcome reporting biases may emerge when there are many analyses that can be performed, but only the analysis with the “best” results is presented resulting in potentially misleading findings [10]. This can take many different representations such as analyzing many different outcomes but reporting only one or some of them, or using different statistical approaches to analyze the same outcome but reporting only one of them. Third, in theory “positive” results may be totally faked, but hopefully such fraud is not common. Overall, these biases ultimately lead to a body of evidence with an inflated proportion of published studies with statistically significant results.

Detecting these biases is not a straightforward process. There are several empirical statistical methods that try to detect publication bias in meta-analyses. The most popular of these are tests of asymmetry, which evaluate whether small or imprecise studies give different results from larger more precise ones [11]. However, these methods may not be very sensitive or specific in the detection of such biases, especially when few studies are included in a meta-analysis [11]–[13].

An alternative approach is the excess significance test. This examines whether too many individual studies in a meta-analysis report statistically significant results compared with what would be expected under reasonable assumptions about the plausible effect size [14]. The excess significance test has low power to detect bias in single meta-analyses with limited number of studies, but a major advantage is its applicability to many meta-analyses across a given field. This increases the power to detect biases that pertain to larger fields and disciplines rather than just single topics. Previous applications have found an excess of statistically significant findings in various human research domains [14]–[17], but it has not been applied to animal research studies.

Biases in animal experiments may result in biologically inert or even harmful substances being taken forward to clinical trials, thus exposing patients to unnecessary risk and wasting scarce research funds. It is important to understand the extent of potential biases in this field, as multiple interventions with seemingly promising results in animals accumulate in its literature. Therefore, in this paper, we probed whether there is evidence for excess statistical significance in animal studies of interventions for neurological diseases using a large database of 160 interventions and 4,445 study datasets.

## Results

### Description of Database

Our database included a total of 4,445 pairwise comparisons from 1,411 unique animal studies that were synthesized in 160 meta-analyses (Table S1). Two meta-analyses (*n* = 1,054 comparisons) pertained to Alzheimer disease (AD), 34 meta-analyses (*n* = 483) to experimental autoimmune encephalomyelitis (EAE), 16 meta-analyses (*n* = 1,403) to focal ischemia, 61 meta-analyses (*n* = 424) to intracerebral hemorrhage (ICH), 45 meta-analyses (*n* = 873) to Parkinson disease (PD), and two meta-analyses (*n* = 208) to spinal cord injury (SCI). The median number of comparisons in each meta-analysis was eight (interquartile range [IQR], 3–23). The median sample size in each animal study dataset was 16 (IQR, 11–20), while the median sample size in each meta-analysis was 135 (IQR, 48–376).

### Summary Effect Sizes

Of the 160 meta-analyses, 112 (70%) had found a nominally (*p*≤0.05) statistically significant summary effect per fixed-effects synthesis, of which 108 meta-analyses favored the experimental intervention and only four meta-analyses favored the control intervention (94 and four for random effects synthesis). The proportion of the associations that had a nominally statistically significant effect using the fixed-effects summary ranged from 57% for ICH to 100% for AD, focal ischemia, and SCI. Table S1 provides information for all 160 meta-analyses. In 47 (29%) meta-analyses the respective most precise study had a nominally statistically significant result, as described in Table 1. The effect size of the most precise study in each meta-analysis was more conservative than the fixed-effects summary in 114 (71%) meta-analyses.

**Table 1:** Description of the 47 meta-analyses where the respective most precise study had a nominally statistically significant effect.

Disease	Outcome	Intervention/Treatment	Cases/Controls	Standardized Mean Difference (95% CI)		
				Most Precise Study^a^	Summary Fixed Effect	Summary Random Effect
EAE	Mean severity	Aminoguanidine	70/82	1·10 (0·19–2·01)	0·40 (0·04–0·77)	0·45 (−0·22 to 1·12)
EAE	Mean severity	Complete freunds adjuvant	44/42	2·79 (1·27–4·31)	1·61 (0·79–2·43)	1·85 (−0·09 to 3·78)
EAE	Mean severity	Estrogen hormone	415/531	0·84 (0·32–1·36)	1·10 (0·91–1·29)	1·49 (1·08–1·90)
EAE	Mean severity	Fingolimod (FTY720)	91/111	1·30 (0·07–2·53)	2·39 (1·75–3·03)	3·14 (1·29–4·98)
EAE	Mean severity	Immunoglobulin	139/90	1·15 (0·67–1·62)	0·89 (0·56–1·23)	1·01 (0·22–1·80)
EAE	Mean severity	Insulin like growth factor	38/38	4·04 (2·29–5·80)	4·85 (3·85–5·85)	5·00 (3·65–6·34)
EAE	Mean severity	MBP	1,138/1,638	0·82 (0·32–1·32)	0·75 (0·65–0·86)	0·98 (0·77–1·19)
EAE	Mean severity	Minocycline	22/25	2·16 (0·90–3·43)	1·68 (0·91–2·45)	1·72 (0·48–2·97)
EAE	Mean severity	Phenytoin	11/11	2·59 (0·68–4·51)	3·15 (1·70–4·59)	3·15 (1·70–4·59)
EAE	Mean severity	Proteolipid protein	19/28	−1·27 (−2·07 to −0·48)	−0·97 (−1·62 to −0·32)	−0·92 (−1·78 to −0·05)
EAE	Mean severity	Prednisolone	17/17	1·55 (0·12–2·98)	1·54 (0·64–2·44)	1·54 (0·64–2·44)
EAE	Mean severity	Rolipram	117/109	0·56 (0·03–1·08)	0·69 (0·41–0·97)	0·63 (0·06–1·19)
Focal ischemia	Infarct volume	Hypothermia	1,803/1,949	1·08 (0·42–1·74)	1·39 (1·30–1·48)	1·69 (1·53–1·86)
Focal ischemia	Infarct volume	Stem Cells	1,611/1,657	0·91 (0·20–1·62)	0·81 (0·71–0·91)	1·21 (1·00–1·41)
Focal ischemia	Infarct volume	Growth factor	981/1,026	0·78 (0·14–1·42)	0·93 (0·82–1·04)	1·09 (0·90–1·27)
Focal ischemia	Infarct volume	Nootropic	120/128	1·44 (0·43–2·44)	0·93 (0·60–1·26)	0·97 (0·53–1·42)
Focal ischemia	Infarct volume	Minocycline	335/337	1·20 (0·55–1·84)	0·84 (0·65–1·03)	0·83 (0·60–1·07)
Focal ischemia	Infarct volume	Melatonin	276/303	1·33 (0·51–2·14)	1·13 (0·90–1·35)	1·13 (0·90–1·35)
Focal ischemia	Infarct volume	NOS donors	280/356	1·58 (0·76–2·41)	0·71 (0·50–0·92)	0·76 (0·44–1·08)
ICH	Neurobehavioural score	Anti-inflammatory	218/214	0·79 (0·10–1·48)	1·19 (0·89–1·48)	2·06 (1·34–2·77)
ICH	Neurobehavioural score	Calcium channel blockers	108/108	1·06 (0·64–1·48)	0·72 (0·42–1·02)	0·47 (−0·04 to 0·98)
ICH	Neurobehavioural score	Growth factor	70/70	4·03 (2·73–5·34)	5·35 (4·55–6·15)	6·37 (4·18–8·55)
ICH	Neurobehavioural score	Tuftsin fragment 1–3	29/26	1·33 (0·59–2·08)	1·21 (0·62–1·79)	1·21 (0·62–1·79)
ICH	Neurobehavioural score	PPAR-gamma agonist	37/37	2·00 (0·94–3·06)	3·36 (2·52–4·20)	5·38 (1·82–8·95)
ICH	Neurobehavioural score	Psychomotor stimulant	98/104	0·76 (0·27–1·25)	0·17 (−0·15 to 0·48)	−0·36 (−1·19 to 0·48)
ICH	Neurobehavioural score	Stem cells	408/371	2·81 (2·09–3·53)	1·64 (1·45–1·84)	1·77 (1·38–2·15)
ICH	Brain water content	Minocycline	12/12	2·33 (0·73–3·94)	2·62 (1·41–3·84)	2·62 (1·41–3·84)
ICH	Brain water content	Growth factor	18/18	5·76 (3·81–7·71)	4·98 (3·51–6·45)	4·95 (3·19–6·70)
ICH	Brain water content	Zinc protoporphyrin	12/12	1·31 (0·01–2·60)	1·40 (0·46–2·34)	1·40 (0·46–2·34)
ICH	Brain water content	Atorvastatin	18/18	3·01 (1·25–4·77)	3·77 (2·28–5·26)	4·12 (1·53–6·71)
ICH	Brain water content	Tuftsin fragment	10/10	1·63 (0·09–3·17)	1·83 (0·69–2·98)	1·83 (0·69–2·98)
ICH	Brain water content	Stem cells	32/32	1·60 (0·43–2·77)	1·43 (0·64–2·22)	1·43 (0·64–2·22)
ICH	Hematoma volume	Recombinant factor VIIa	12/12	−1·74 (−3·15 to −0·32)	−2·03 (−3·11 to −0·96)	−2·03 (−3·11 to −0·96)
ICH	Hematoma volume	Tuftsin fragment	12/12	1·35 (0·15–2·56)	1·57 (0·59–2·54)	1·57 (0·59–2·54)
ICH	Hematoma volume	Rosiglitazone	11/11	2·05 (0·07–4·02)	3·01 (1·59–4·44)	3·03 (1·06–5·01)
PD	Various	A86929	68/68	2·31 (0·20–4·42)	1·55 (0·76–2·34)	1·55 (0·76–2·34)
PD	Rotational behavior	Aplindore	105/105	0·78 (0·04–1·53)	1·73 (1·40–2·06)	1·88 (1·25–2·51)
PD	Various	Ciladopa (AY27110)	100/100	1·40 (0·55–2·25)	0·002 (−0·38 to 0·38)	−0·37 (−1·36 to 0·62)
PD	Various	Bromocriptine	436/490	0·97 (0·02–1·91)	0·96 (0·76–1·17)	0·95 (0·71–1·18)
PD	Rotational behavior	Dinapsoline	131/131	1·47 (0·07–2·87)	2·03 (1·59–2·47)	2·30 (1·48–3·11)
PD	Various	Lisuride	100/146	1·04 (0·18–1·90)	1·39 (0·99–1·80)	1·39 (0·99–1·80)
PD	Various	Pramipexole	146/143	1·81 (0·45–3·16)	1·42 (0·97–1·87)	1·43 (0·83–2·03)
PD	Various	Quinelorane	24/21	2·48 (0·88–4·07)	1·06 (0·15–1·97)	1·22 (−0·43 to 2·86)
PD	Various	Quinpirole	320/312	1·47 (0·77–2·17)	1·09 (0·87–1·31)	1·08 (0·65–1·50)
PD	Various	Rotigotine	75/83	1·81 (0·88–2·74)	1·76 (1·21–2·32)	1·76 (1·21–2·32)
PD	Various	SKF 80723	10/10	2·30 (0·51–4·09)	3·04 (1·42–4·67)	4·04 (−0·02 to 8·09)
PD	Various	Talipexole	239/238	1·03 (0·33–1·73)	0·79 (0·51–1·07)	0·86 (0·45–1·27)

a Standardized mean difference and 95% CI of the most precise study (smallest standard error) in each meta-analysis.

PPAR, peroxisome proliferator-activated receptor; A86929, a dopamine receptor agonist; SKF 80723, a benzazepine D1 dopamine agonist.

### Between-Study Heterogeneity

There was statistically significant heterogeneity at *p*≤0.10 for 83 (52%) meta-analyses (Table S1). There was moderate heterogeneity (I^2^ = 50%–75%) in 52 (33%) meta-analyses, and high heterogeneity (I^2^>75%) in 22 (14%). The lowest proportion of significant heterogeneity was observed in meta-analyses of ICH (36%) and PD (42%), while all other areas had significant heterogeneities above 70%. Uncertainty around the heterogeneity estimates was often large, as reflected by wide 95% CI of I^2^.

### Small-Study Effects

There was evidence of small-study effects in 74 (46%) meta-analyses (Table S1). These pertained to AD (*n* = 2 meta-analyses), EAE (*n* = 14), focal ischemia (*n* = 9), ICH (*n* = 27), PD (*n* = 21), and SCI (*n* = 1).

### Excess of Significance

When the plausible effect was assumed to be that of the most precise study in each meta-analysis, there was evidence (*p*≤0.10) of excess significance in 49 (31%) meta-analyses (AD *n* = 2, EAE *n* = 13, focal ischemia *n* = 11, ICH *n* = 10, PD *n* = 12, SCI *n* = 1) (Table 2), despite the generally low power of the excess significance test. Under the assumptions of the summary fixed effect being the plausible effect, there was evidence of excess significance in 23 meta-analyses.

**Table 2:** Observed and expected number of “positive” studies in the 49 meta-analyses with a significant excess of “positive” studies under the assumption that the plausible effect size equals the effect of the most precise study in each meta-analysis.

Disease	Outcome	Intervention/Treatment	Studies	Comparisons	Observed Positive	Most Precise Study Effect		Summary Fixed Effect	
						Expected Positive^a^	*p*-Value*	Expected Positive^b^	*p*-Value*
AD	Morris water maze	Various	82	250	76	45·8	3·8 • 10^−6^	51·3	2·2 • 10^−4^
AD	Plaque area	Various	318	804	342	40·6	<1 • 10^−9^	226	<1 • 10^−9^
EAE	Mean severity	Bone marrow	2	24	15	1·2	<1 • 10^−9^	14·5	0·99
EAE	Mean severity	Cobra venom factor	2	8	4	1·17	0·02	3·95	0·99
EAE	Mean severity	Cyclosporin	8	14	6	3·05	0·10	4·43	0·39
EAE	Mean severity	Estrogen hormone	7	42	22	15·8	0·06	22·3	NP
EAE	Mean severity	Glatiramer acetate	9	20	13	2·4	4 • 10^−8^	11·5	0·65
EAE	Mean severity	Interleukin 10	7	34	9	4·6	0·04	2·15	2 • 10^−4^
EAE	Mean severity	Interleukin 4	3	10	2	0·52	0·09	1·51	0·65
EAE	Mean severity	Lovastatin	2	7	4	0·93	7·7 • 10^−3^	4·59	NP
EAE	Mean severity	Methylprednisolone	4	10	6	3·15	0·08	0·69	1·8 • 10^−5^
EAE	Mean severity	Mitoxantrone	4	30	20	5·29	<1 • 10^−9^	25·4	NP
EAE	Mean severity	Rolipram	3	9	7	2·16	1 • 10^−3^	2·99	8·2 • 10^−3^
EAE	Mean severity	Spinal cord protein	2	5	2	0·32	0·04	0·34	0·04
EAE	Mean severity	Vitamin D	6	13	9	1·88	1·1 • 10^−5^	9·19	NP
Focal ischemia	Infarct volume	Hypothermia	107	221	135	101	7·2 • 10^−6^	145	NP
Focal ischemia	Infarct volume	Stem cells	110	211	84	67·6	0·02	63·7	3·3 • 10^−3^
Focal ischemia	Infarct volume	Thrombolytic	83	202	60	10·2	<1 • 10^−9^	41·1	1·6 • 10^−3^
Focal ischemia	Infarct volume	Anti-oxidants	18	34	14	6·31	2·8 • 10^−3^	13·2	0·86
Focal ischemia	Infarct volume	Growth factor	78	128	53	32·7	9·7 • 10^−5^	42·7	0·06
Focal ischemia	Infarct volume	Anti-inflammatory	23	44	31	2·36	<1 • 10^−9^	20	1·2 • 10^−3^
Focal ischemia	Infarct volume	Estrogens	33	100	46	10·4	<1 • 10^−9^	25·6	9·7 • 10^−6^
Focal ischemia	Infarct volume	Urokinase	12	26	18	2·26	<1 • 10^−9^	10·1	2 • 10^−3^
Focal ischemia	Infarct volume	NXY-059	10	29	14	1·54	<1 • 10^−9^	11·1	0·26
Focal ischemia	Infarct volume	NOS inhibitors	65	148	63	34·6	2·6 • 10^−7^	33	5 • 10^−8^
Focal ischemia	Infarct volume	Tacrolimus (FK506)	29	95	55	15·8	<1 • 10^−9^	50·3	0·36
ICH	Neurobehavioural score	Minocycline	3	9	3	0·45	8·5 • 10^−3^	0·46	9 • 10^−3^
ICH	Neurobehavioural score	Anti-inflammatory	7	26	15	7·32	1·7 • 10^−3^	13·6	0·70
ICH	Neurobehavioural score	Anti-oxidants	9	32	12	1·6	2 • 10^−8^	2·03	3 • 10^−7^
ICH	Neurobehavioural score	Atorvastatin	2	6	3	0·31	2·5 • 10^−3^	2·97	0·99
ICH	Neurobehavioural score	Iron chelator	6	14	7	1·9	1·2 • 10^−3^	6·35	0·79
ICH	Brain water content	Anti-inflammatory	7	13	4	1·04	0·02	4·05	NP
ICH	Brain water content	Anti-oxidants	5	29	9	3·12	2·6 • 10^−3^	2·15	1·7 • 10^−4^
ICH	Brain water content	Deferoxamine	2	12	6	1·05	2·6 • 10^−4^	5·58	0·99
ICH	Brain water content	Estradiol	2	4	2	0·28	0·03	1·06	0·29
ICH	Hematoma volume	Anti-inflammatory	5	8	2	0·4	0·06	0·84	0·2
PD	Various	PHNO	15	41	6	2·05	0·02	14·8	NP
PD	Various	7-OH-DPAT	4	7	4	0·8	4·4 • 10^−3^	4·12	NP
PD	Rotational behavior	Aplindore	4	7	7	3·49	7·7 • 10^−3^	6·99	0·99
PD	Various	Apomorphine	54	156	63	32·2	2 • 10^−8^	30·3	<1 • 10^−9^
PD	Various	Cabergoline	6	14	4	0·91	0·01	1·9	0·11
PD	Various	Pergolide	8	14	4	1·3	0·03	3·25	0·75
PD	Various	Piribedil	12	42	5	2·17	0·06	10·4	NP
PD	Various	Ropinirole	13	33	9	4·22	0·03	19·1	NP
PD	Various	S32504	15	36	14	2·5	5 • 10^−8^	12·7	0·73
PD	Various	SKF 83959	3	4	2	0·39	0·05	0·96	0·25
PD	Various	SKF 38393	14	38	12	6·33	0·03	5·62	9·2 • 10^−3^
PD	Various	SKF 82958	10	16	7	2·12	2·7 • 10^−3^	5·73	0·6
SCI	Neurobehavior-motor	Stem cells	89	186	85	16·5	<1 • 10^−9^	85·8	NP

a Expected number of statistically significant studies using the effect of the most precise study of each meta-analysis as the plausible effect size.

b Expected number of statistically significant studies using the summary fixed effects estimate of each meta-analysis as the plausible effect size.

* *p*-value of the excess statistical significance test· All statistical tests were two-sided.

7-OH-DPAT, a dopamine D3 receptor agonist; NP, not pertinent, because the estimated E is larger than the O, and there is no evidence of excess statistical significance based on the assumption made for the plausible effect size; NXY-059, disufenton sodium; PHNO, a dopamine D2 receptor agonist; S32504, a dopamine D3/D2 receptor agonist; SKF 82958, a benzazepine D1/D5 dopamine agonist; SKF 38393, a benzazepine D1/D5 dopamine agonist; SKF 83959, a benzazepine D1/D2 dopamine agonist.

When the excess of significance was examined in aggregate across all 4,445 studies (Table 3), excess significance was present when assuming as plausible effect the effect of the most precise study (*p*<1.10^−9^). The observed number of “positive” studies was O = 1,719, while the expected was E = 919. Excess significance was also documented in studies of each of the six disease categories. An excess of “positive” studies was observed also when assuming the summary fixed effect as the plausible effect size (*p*<1.10^−9^).

**Table 3:** Observed and expected number of “positive” studies by type of neurological disease.

Disease	Comparisons	Observed Positive	Most Precise Study Effect		Summary Fixed Effect	
			Expected Positive^a^	*p*-Value*	Expected Positive^b^	*p*-Value*
All	4,445	1,719	919	<1 • 10^−9^	1,450	<1 • 10^−9^
AD	1,054	418	86·4	<1 • 10^−9^	277	<1 • 10^−9^
EAE	483	212	131	<1 • 10^−9^	187	0·02
Focal ischemia	1,403	626	370	<1 • 10^−9^	517	<1 • 10^−9^
ICH	424	145	108	5·6 • 10^−5^	130	0·11
PD	873	228	202	0·04	254	NP
SCI	208	90	21·6	<1 • 10^−9^	90·3	NP

a Expected number of statistically significant studies using the effect of the most precise study of each meta-analysis as the plausible effect size.

b Expected number of statistically significant studies using the summary fixed effects estimate of each meta-analysis as the plausible effect size.

* *p*-Value of the excess statistical significance test. All statistical tests were two-sided.

NP, not pertinent, because the estimated E is larger than the O, and there is no evidence of excess statistical significance based on the assumption made for the plausible effect size.

Similar results were observed in analyses according to methodological or reporting characteristics of included studies (Table 4). Under the assumption of the effect of the most precise study being the plausible effect, there was evidence of excess significance in all subgroups. However, the strongest excesses of significance (as characterized by the ratio of O over E) were recorded specifically in meta-analyses where small-study effects had also been documented (O/E = 2.94), in those meta-analyses with the least precise studies (O/E = 2.94 in the bottom quartile of weight), and in those meta-analyses where the corresponding studies included a statement about the presence of conflict of interest (O/E = 3.27). Under the assumption of the summary fixed effects being the plausible effect size, excess significance was still formally documented in the large majority of subgroups, but none had such extreme O/E ratios (Table 4).

**Table 4:** Observed and expected number of “positive” studies for all neurological diseases in subgroups.

Subgroup	Studies	Observed Positive (O)	Most Precise Study Effect			Summary Fixed Effect		
			Expected Positive (E)^a^	O/E Ratio	*p*-Value*	Expected Positive (E)^b^	O/E Ratio	*p*-Value*
Heterogeneity								
I^2^≤50%	1,098	264	217	1·22	5.6 • 10^−4^	317	0·83	NP
I^2^>50%	3,347	1,455	702	2·07	<1 • 10^−9^	1,140	1·28	<1 • 10^−9^
Small-study effects								
Yes	2,773	1,139	387	2·94	<1 • 10^−9^	953	1·20	<1 • 10^−9^
No	1,672	580	532	1·09	0·01	501	1·16	3·5 • 10^−5^
Meta-analysis (fixed-effect)								
Significant	4,055	1,644	871	1·89	<1 • 10^−9^	1,420	1·16	<1 • 10^−9^
Non-significant	390	75	47·5	1·58	6·9 • 10^−5^	30·2	2·48	<1 • 10^−9^
Weight (1/SE^2^) of the most precise study^c^								
Q1	1,275	553	188	2·94	<1 • 10^−9^	422	1·31	<1 • 10^−9^
Q2	1,051	402	204	1·97	<1 • 10^−9^	293	1·37	<1 • 10^−9^
Q3	1,032	424	229	1·85	<1 • 10^−9^	350	1·21	1·8 • 10^−6^
Q4	1,087	340	298	1·14	4·7 • 10^−3^	390	0·87	NP
Random allocation								
Yes	994	415	200	2·08	<1 • 10^−9^	346	1·20	6·7 • 10^−6^
No	3,185	1,207	641	1·88	<1 • 10^−9^	1,020	1·18	<1 • 10^−9^
Blinded induction								
Yes	208	74	42·6	1·74	4·4 • 10^−7^	79·4	0·93	NP
No	2,615	1,108	465	2·38	<1 • 10^−9^	845	1·31	<1 • 10^−9^
Blinded outcome								
Yes	1,236	482	230	2·10	<1 • 10^−9^	418	1·15	1·3 • 10^−4^
No	2,943	1,140	610	1·87	<1 • 10^−9^	947	1·20	<1 • 10^−9^
Sample size calculation								
Yes	39	8	3·87	2·07	0·05	14·9	0·54	NP
No	4,140	1,614	837	1·93	<1 • 10^−9^	1,350	1·20	<1 • 10^−9^
Animal welfare compliance								
Yes	2,436	943	467	2·02	<1 • 10^−9^	805	1·17	<1 • 10^−9^
No	1,743	679	373	1·82	<1 • 10^−9^	559	1·21	<1 • 10^−9^
Conflict of interest								
Yes	319	160	49·2	3·25	<1 • 10^−9^	109	1·47	1 • 10^−8^
No	3,860	1,462	791	1·85	<1 • 10^−9^	1,260	1·16	<1 • 10^−9^

a Expected number of statistically significant studies using the effect of the most precise study of each meta-analysis as the plausible effect size.

b Expected number of statistically significant studies using the summary fixed effects estimate of each meta-analysis as the plausible effect size.

* *p*-Value of the excess statistical significance test· All statistical tests were two-sided.

c Quartiles of the weight of the most precise study in each meta-analysis.

NP, not pertinent, because the estimated E is larger than the O, and there is no evidence of excess statistical significance based on the assumption made for the plausible effect size.

### Interventions with Strong Evidence of Association

**Figure 1:**
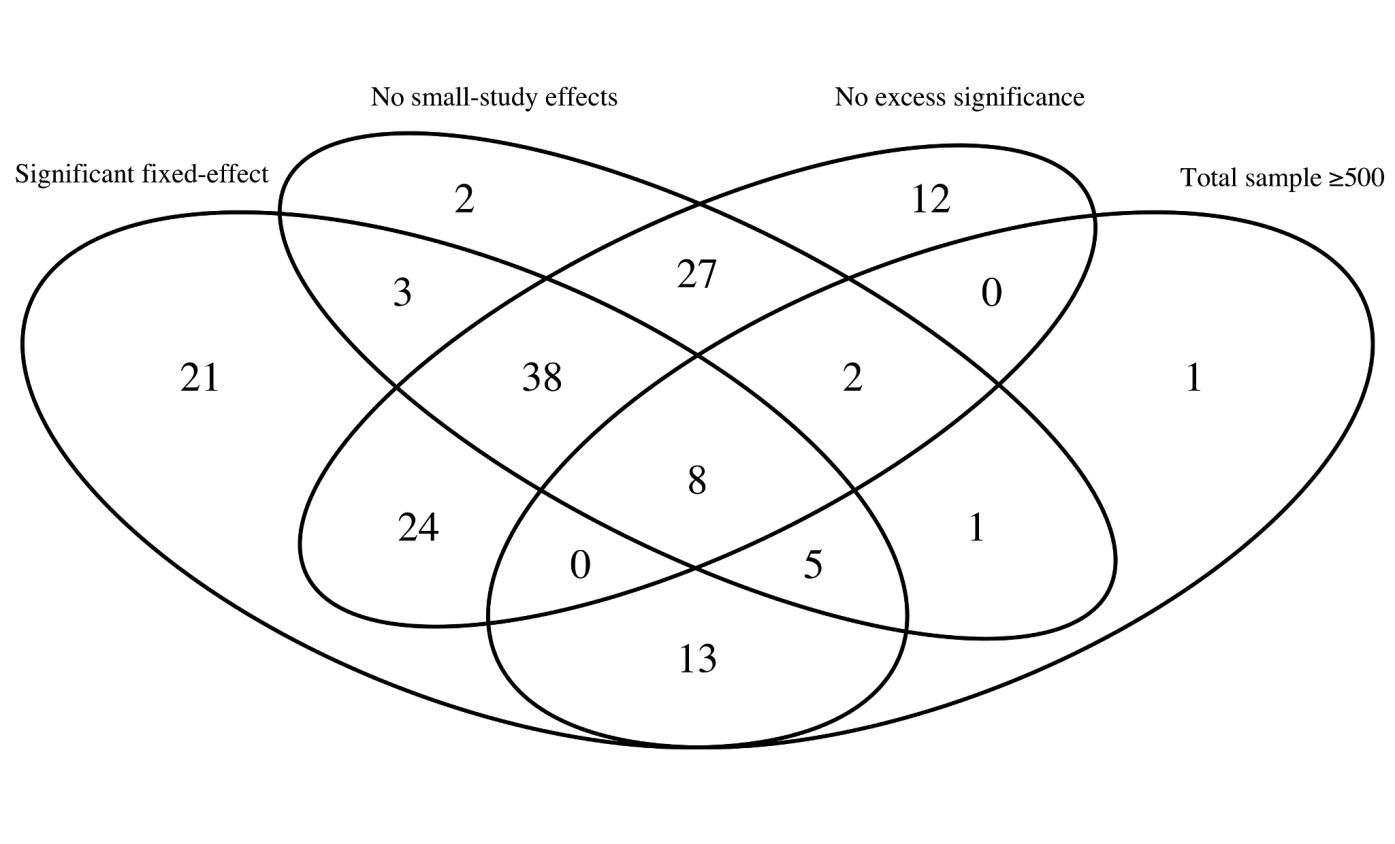
Venn diagrams of the meta-analyses of animal studies of neurological disorders. We plotted the number of studies with a total sample size of at least 500 animals; those which showed a nominally (*p*≤0.05) statistically significant effect per fixed-effects synthesis; those that had no evidence of small-study effects; and those that had no evidence of excess significance. The numbers represent the studies that have two or more of the above characteristics according to the respective overlapping areas.

Only 46 meta-analyses (29%) found interventions with a nominally significant effect per fixed-effects synthesis and no evidence of small-study effects or excess significance (when this calculation was based on the plausible effect being that of the most precise study) (Figure 1). Of those, only eight had a total sample size of over 500 animals: one pertained to EAE (myelin basic protein [MBP]), four pertained to focal ischemia (minocycline, melatonin, nicotinamide, nitric oxide species [NOS] donors), one pertained to ICH (stem cells), and two to PD (bromocriptine, quinpirole).

## Discussion

We evaluated 160 meta-analyses of animal studies describing six neurological conditions, most of which had found a nominally (*p*≤0.05) statistically significant fixed-effects summary favoring the experimental intervention. The number of nominally statistically significant results in the component studies of these meta-analyses was too large to be true, and this evidence of excess significance was present in studies across all six neurological diseases. Overall, only eight of the 160 meta-analyses had nominally significant results, no suggestion of bias related to small-study effects or excess of significant findings, and evidence procured from over 500 animals.

Animal studies represent a considerable proportion of the biomedical literature with approximately five million papers indexed in PubMed [8]. These studies are conducted to do a first-pass evaluation of the effectiveness and safety of therapeutic interventions. However, there is great discrepancy between the intervention effects found in preclinical animal studies and those found in clinical trials of humans with most of these interventions rarely achieving successful translation [2],[3],[18]. Possible explanations for this failure include differences in the underlying biology and pathophysiology between humans and animals, but also the presence of biases in study design or reporting of the animal literature.

Our empirical evaluation of animal studies on neurological disorders found a significant excess of nominally statistically significant studies, which suggests the presence of strong study design or reporting biases. Prior evaluations of animal studies had also noted that alarmingly the vast majority of the published studies had statistically significant associations, and had suggested high prevalence of publication bias [9],[19], resulting in spurious claims of effectiveness. We observed excessive nominally significant results in all subgroup categories defined by random allocation of treatment, blinded induction of treatment, blinded assessment of the outcome, sample size calculation, or compliance to animal welfare. This suggests that the excess of significance in animal studies of neurological disorders may reflect reporting biases that operate regardless of study design features. It is nevertheless possible that reporting biases are worst in fields with poor study quality, although this was not clear in our evaluation. Deficiencies in random allocation and blinded induction of the treatment or blinded assessment of the outcome have been associated with inflated efficacy estimates in other evaluations of animal research [20],[21].

We also documented very prominent excess of significant results (observed “positive” results being three times the number of expected) for interventions that had also evidence of small-study effects and in meta-analyses with the least precise studies. Both of these observations are commensurate with reporting bias being the explanation for the excess significance, with bias being more prominent in smaller studies and becoming more discernible when sufficiently precise studies are also available.

Conventional publication bias (non-publication of neutral/negative results), may exist in the literature of animal studies on neurological disorders. Our evaluation showed that 46% of the meta-analyses had evidence of small-study effects, which may signal publication bias. However, this association is not specific, and the Egger test used to evaluate small-study effects is underpowered especially when it evaluates few and small studies in a meta-analysis [13]. It is also likely that selective outcome or analysis reporting biases exist. The animal studies on neurological disorders used many different outcomes and methods to measure each outcome as can be seen across the Table S1, and they may have used different statistical analysis techniques and applied several different rules for inclusion and exclusion of data. Thus, it is possible that individual studies may have measured different outcomes, tested a variety of inclusion and exclusion criteria and performed several statistical analyses, but reported only a few findings guided in part by the significance of the results. Detection of such biases is difficult and no formal well-developed statistical test exists. Evidence is usually indirect and requires access to the study protocol or even communication with the investigators.

In contrast to the above, we found eight interventions with strong and statistically significant benefits in animal models and without evidence of small-study effects or excess significance. However, the data for these interventions may still have compromised internal validity; having identified one of these, melatonin, as a candidate treatment for stroke, we tested efficacy in an animal study designed specifically to avoid some of the prevalent sources of bias. Under these circumstances melatonin had no significant effect on outcome [22].

It is interesting to discuss whether human experimental evidence for these interventions is more promising than the generally disappointing results seen for most interventions that have previously given some signal of effectiveness in animals. A meta-analysis of 33 animal studies showed that administration of MBP reduced the severity of EAE, which is an animal model for multiple sclerosis. However, a phase III randomized clinical trial (RCT) in humans showed no significant differences between MBP and placebo [23]. Minocycline, a tetracycline antibiotic with potential neuroprotective effects, showed improvements in stroke scales in two human RCTs, but these were small phase II trials [24],[25] and have not been confirmed in larger studies. Several animal studies of melatonin, an endogenously produced antioxidant, have reported a beneficial effect on infarct volume [26], but RCTs with clinical endpoints in humans don't exist. A small RCT did not show significant differences in oxidative or inflammatory stress parameters between melatonin and placebo [27]. Administration of nicotinamide, the amide of vitamin B3, to animals with focal ischemia reduced the infarct volume [19], but RCTs have not evaluated clinical outcomes in relation to nicotinamide [28]. Several animal studies of NOS donors, like the anti-anginal drug nicorandil, have shown reductions in infarct volume, and RCTs have also shown that nicorandil improves cardiac function in patients with acute myocardial infarction [29],[30]. Granulocyte-colony stimulating factor (G-CSF), a growth factor that stimulates the bone marrow to produce granulocytes and stem cells, has been reported in animal studies to improve the neurobehavioral score in animals with ICH. Some similar evidence exists from RCTs of stroke patients, but it consists of small phase II trials [31],[32], and an unpublished phase III trial was neutral (Ringelstein P et al., International Stroke Conference, Feb 2012). Bromocriptine and quinpirole are dopamine agonists that have been successfully used in animal studies of PD [33]. Bromocriptine is approved to treat PD in humans [34], but no human trial exists for quinpirole. In spite of this patchy record, interventions with strong evidence of efficacy and no hints of bias in animals may be prioritized for further testing in humans.

Some limitations should be acknowledged in our work. First, asymmetry and excess significance tests offer hints of bias, not definitive proof thereof. Most individual animal studies were small with a median total sample size of 16 animals, and a median number of eight comparisons in each meta-analysis. Therefore, the interpretation of the excess significance test for the results of a single meta-analysis should be very cautious. A negative test for excess significance does not exclude the potential for bias [14]. The most useful application of the excess significance test is to give an overall impression about the average level of bias affecting the whole field of animal studies on neurological disorders.

Second, the exact estimation of excess statistical significance is influenced by the choice of plausible effect size. We performed analyses using different plausible effect sizes, including the effect of the most precise study in each meta-analysis, and the summary fixed effect; these yielded similar findings. Effect inflation may affect even the results of the most precise studies, since often these were not necessarily very large or may have had inherent biases themselves, or both. Thus, our estimates of the extent of excess statistical significance are possibly conservative, and the problem may be more severe.

Third, we evaluated a large number of meta-analyses on six neurological conditions, but our findings might not necessarily be representative of the whole animal literature. However, biases and methodological deficits have been described for many animal studies regardless of disease domain [1],[2],[21].

In conclusion, the literature of animal studies on neurological disorders is probably subject to considerable bias. This does not mean that none of the observed associations in the literature are true. For example, we showed evidence of eight strong and statistically significant associations without evidence of small-study effects or excess significance. However, only two (NOS donors and focal ischemia, and bromocriptine and PD) of even these eight associations seem to have convincing RCT data in humans. We support measures to minimize bias in animal studies and to maximize successful translation into human applications of promising interventions [35]. Study design, conduct, and reporting of animal studies can be improved by following published guidelines for reporting animal research [35],[36]. Publication and selective reporting biases may be diminished by preregistering experimental animal studies. Access to the study protocol and also to raw data and analyses would allow verification of their results, and make their integration with other parallel or future efforts easier. Systematic reviews and meta-analyses and large consortia conducting multi-centre animal studies should become routine to ensure the best use of existing animal data, and to aid in the selection of the most promising treatment strategies to enter human clinical trials.

## Materials and Methods

### Study Identification

We used data from published and unpublished meta-analyses of interventions tested in animal studies of six neurological diseases (AD, EAE, focal ischemia, ICH, PD, and SCI) deposited in the database of Collaborative Approach to Meta-Analysis and Review of Animal Data in Experimental Studies (CAMARADES), which is an international collaboration established in 2004 with an aim to support meta-analyses of animal data [20]. The database is representative of the literature, and includes details of each individual experiment. Out of the total of 14 published meta-analyses of animal stroke studies, 11 are part of the CAMARADES database. Animal studies report a variety of outcome measures often measured from the same cohort of animals. To ensure independence, we only used one outcome analysis per animal cohort. Where multiple outcomes were reported from a single cohort, we chose the one that was most frequently found in each meta-analysis. We abstracted the following information from each study: publication year, intervention, outcome, animal cohort, effect size and standard error, number of affected animals in the treatment and control group, and ten binary methodological criteria (peer-reviewed publication, statement of control of temperature, random allocation to treatment or control, blinded induction of treatment, blinded assessment of outcome, use of anesthetic without significant intrinsic neuroprotective activity, appropriate animal model, sample size calculation, compliance with animal welfare regulations, and statement of potential conflict of interest), which were based on relevant guidelines for reporting animal research [35]–[38].

### Estimation of Summary Effect and Heterogeneity

For each meta-analysis, we estimated the summary effect size and its confidence intervals using both fixed and random effect models [39]. All outcomes included in the database were measured on a continuous scale, and hence we used the standardized mean difference as the effect size. We also tested for between-study heterogeneity estimating the *p*-value of the χ^2^-based Cochran Q test, and the I^2^ metric of inconsistency. Q is obtained by the weighted sum of the squared differences of the observed effect in each study minus the fixed summary effect [40]. I^2^ ranges from 0% to 100% and describes the percentage of variation across studies that is attributed to heterogeneity rather than chance [41]. The corresponding 95% CIs were also calculated [42].

### Asymmetry Tests for Small-Study Effects

We evaluated whether there is evidence for small-study effects, i.e., whether smaller studies give substantially different estimates of effect size compared to larger studies. This may offer a hint for publication or other selective reporting biases, but they may also reflect genuine heterogeneity, chance or other reasons for differences between small and large studies [11]. We applied the regression asymmetry test proposed by Egger et al [43]. A *p*≤0.10 with more conservative effect in larger studies was considered evidence for small-study effects.

### Evaluation of Excess Significance

We applied the excess significance test, which is an exploratory test that evaluates whether there is a relative excess of formally significant findings in the published literature due to any reason. This test evaluates whether the observed number of studies (O) with nominally statistically significant results (“positive” studies, *p*≤0.05) within a meta-analysis differs from their expected number (E). “Positive” findings were counted in both directions, i.e., when the experimental intervention is more beneficial compared to the control, as well as when the experimental intervention is harmful. If there is no excess significance bias, then O = E. The greater the difference between O and E, the greater is the extent of excess significance bias.

We used a binomial test, as previously presented in detail [14]. This test evaluates whether the number of “positive” studies, among those in a meta-analysis, is too large based on the power that these studies have to detect plausible effects at α = 0.05. The O versus E comparison is performed separately for each meta-analysis, and it is also extended to many meta-analyses after summing O and E across meta-analyses.

E is calculated in each meta-analysis by the sum of the statistical power estimates for each component study. The estimated power of each component study depends on the plausible effect size for the tested animal study association. The true effect size for any meta-analysis is not known. We performed the main analysis using the effect size of the most precise study (with the smallest standard error) in a meta-analysis as the plausible effect. The estimate from this most precise study, other things being equal, should be closer to the true estimate than the results of less precise studies, especially if biases affect predominantly the literature of smaller studies (small-study effects) [44]–[46]. Additionally, we conducted sensitivity analysis using as the plausible effect size the fixed effects summary from each meta-analysis. In the presence of bias these summary fixed effects may be larger than the true effects, and this situation may arise even for the effect estimate of the most precise study, albeit to a smaller extent. Therefore, all these assumptions tend to be conservative in testing for excess significance. We did not use the random effects summary, because it tends to be overtly inflated in the presence of small-study effects, as the small studies receive increased relative weight in random effects calculations [47].

The power of each study was calculated using the Wilcoxon rank sum test under the family of Lehmann alternative hypotheses in a freely available software [48], which is an exact calculation suitable for the very small sample sizes that are typical of many animal studies [49]. Excess significance for single meta-analyses was claimed at *p*≤0.10 (one-sided *p*≤0.05 with O>E as previously proposed), since the test is expected to have low power to detect a specific excess of significant findings especially when there are a few “positive” studies [14].

We assessed excess significance in aggregate across all six neurological diseases, and separately in each one of them, as selective reporting bias may affect different research domains to a different extent. These domains may also have different typical magnitudes of effect sizes for the tested interventions, and different analytical biases even if research is sometimes conducted by the same teams across various domains.

The excess significance test was also performed in subgroups: for meta-analyses with I^2^≤50% and >50%, as values exceeding 50% are typically considered evidence of large heterogeneity beyond chance [50]; by evidence or not of small-study effects as per Egger's test; by whether the summary fixed effect of the respective meta-analysis was nominally statistically significant or not; by quartiles of the weight of the most precise study for each meta-analysis; by use of random allocation of the treatment or not; by use of blinded induction of the treatment or not; by use of blinded assessment of the outcome or not; by whether the sample size calculation was described or not; by whether the study reported to comply to the animal welfare regulations or not; and by whether potential conflicts of interest were reported or not.

## Supporting Information

Table S1 **Analytical description of the 160 meta-analyses with observed and expected numbers of “positive” study datasets.**
(XLSX)

## Abbreviations

AD: Alzheimer disease
CAMARADES: Collaborative Approach to Meta-Analysis and Review of Animal Data in Experimental Studies
E: number of expected studies with statistically significant results
EAE: experimental autoimmune encephalomyelitis
ICH: intracerebral hemorrhage
IQR: interquartile range
MBP: myelin basic protein
NOS: nitric oxide species
O: number of observed studies with statistically significant results
PD: Parkinson disease
RCT: randomized clinical trial
SCI: spinal cord injury
